# Alzheimer Disease: Crosstalk between the Canonical Wnt/Beta-Catenin Pathway and PPARs Alpha and Gamma

**DOI:** 10.3389/fnins.2016.00459

**Published:** 2016-10-19

**Authors:** Alexandre Vallée, Yves Lecarpentier

**Affiliations:** ^1^CHU Amiens Picardie, Université Picardie Jules VerneAmiens, France; ^2^Experimental and Clinical Neurosciences Laboratory, INSERM U1084, University of PoitiersPoitiers, France; ^3^AP-HP, Epidemiology and Clinical Research Department, University Hospital Bichat-Claude BernardParis, France; ^4^Centre de Recherche Clinique, Hôpital de MeauxMeaux, France

**Keywords:** PPAR alpha, PPAR gamma, Wnt/beta-catenin, Alzheimer disease, lithium, riluzole, glycogen synthase kinase-3beta

## Abstract

The molecular mechanisms underlying the pathophysiology of Alzheimer's disease (AD) are still not fully understood. In AD, Wnt/beta-catenin signaling has been shown to be downregulated while the peroxisome proliferator-activated receptor (PPAR) gamma (mARN and protein) is upregulated. Certain neurodegenerative diseases share the same Wnt/beta-catenin/PPAR gamma profile, such as bipolar disorder and schizophrenia. Conversely, other NDs share an opposite profile, such as amyotrophic lateral sclerosis, Parkinson's disease, Huntington's disease, multiple sclerosis, and Friedreich's ataxia. AD is characterized by the deposition of extracellular Abeta plaques and the formation of intracellular neurofibrillary tangles in the central nervous system (CNS). Activation of Wnt signaling or inhibition of both glycogen synthase kinase-3beta and Dickkopf 1, two key negative regulators of the canonical Wnt pathway, are able to protect against Abeta neurotoxicity and to ameliorate cognitive performance in AD patients. Although PPAR gamma is upregulated in AD patients, and despite the fact that it has been shown that the PPAR gamma and Wnt/beta catenin pathway systems work in an opposite manner, PPAR gamma agonists diminish learning and memory deficits, decrease Abeta activation of microglia, and prevent hippocampal and cortical neurons from dying. These beneficial effects observed in AD transgenic mice and patients might be partially due to the anti-inflammatory properties of PPAR gamma agonists. Moreover, activation of PPAR alpha upregulates transcription of the alpha-secretase gene and represents a new therapeutic treatment for AD. This review focuses largely on the behavior of two opposing pathways in AD, namely Wnt/beta-catenin signaling and PPAR gamma. It is hoped that this approach may help to develop novel AD therapeutic strategies integrating PPAR alpha signaling.

## Introduction

Alzheimer disease (AD) represents the most common form of neurodegenerative dementia. The AD pathophysiology is not completely understood but is characterized by the deposition of extracellular Abeta plaques (Abeta) and the formation of intracellular neurofibrillary tangles (NFTs) in the central nervous system (CNS) (Mattson, 2004; Mayeux and Stern, 2012). Certain neurodegenerative diseases (NDs) have recently been classified into two categories (Lecarpentier et al., 2014). Amyotrophic lateral sclerosis (Lecarpentier and Vallee, 2016), Parkinson's disease, Huntington's disease, multiple sclerosis and Friedreich's ataxia represent NDs in which the canonical Wnt/beta-catenin pathway is upregulated while PPAR gamma is downregulated. Conversely, AD, bipolar disorder and schizophrenia are NDs in which the canonical Wnt/beta-catenin pathway is downregulated while PPAR gamma is upregulated. Thus, stimulating Wnt/beta-catenin signaling could represent a promising therapeutic target for human AD treatment, since its activation protects neurons against Abeta toxicity, the hallmark of the disease (Mao et al., 2001; Alvarez et al., 2004; Boonen et al., 2009; Fiorentini et al., 2010; Shruster et al., 2011; Zhang et al., 2011; Inestrosa et al., 2012, 2015). Importantly, PPAR gamma levels (mRNA and protein) have been found to be elevated in AD brain tissues (Kitamura et al., 1999; de la Monte and Wands, 2006). Although PPAR gamma expression is high in AD, PPAR gamma agonists have been used in AD for both humans and animal models and have been shown to induce beneficial effects (Combs et al., 2000; Sastre et al., 2003; Camacho et al., 2004; D'Abramo et al., 2005; Pedersen et al., 2006; Risner et al., 2006; Escribano et al., 2010).

Given that in numerous tissues and pathological states PPAR gamma activation induces repression of the Wnt/beta-catenin pathway (Moldes et al., 2003; Sharma et al., 2004; Liu et al., 2006), the rationale for using PPAR gamma in AD would seem to merit discussion. The anti-inflammatory properties induced by PPAR gamma agonists may partly explain their beneficial therapeutic effects. Compared with PPAR gamma, PPAR alpha has been poorly studied in AD. PPAR alpha levels (mRNA and protein) have been found to be low in AD brain tissues (de la Monte and Wands, 2006) and a recent study highlights the interest of studying PPAR alpha signaling in AD (Corbett et al., 2015). In numerous diseases, PPAR gamma expression varies in an opposite way to that of both PPAR alpha and Wnt signaling. The links between Wnt/beta-catenin signaling and PPARs alpha and gamma in AD are reviewed here.

## The canonical Wnt/beta-catenin pathway

**Figure 1:**
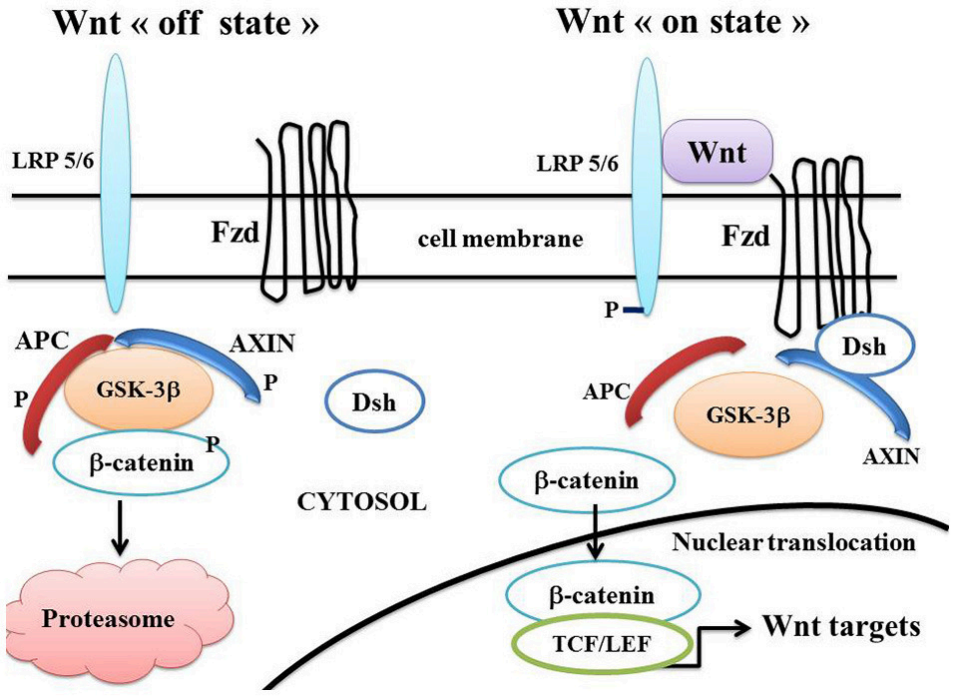
**A schematic model of the Wnt/beta-catenin pathway**. In the Wnt “on state” (right part), Wnt binds both Fzd and LRP5/6 receptors to initiate LRP phosphorylation as well as Dsh/Fzd internalization. Dsh membrane translocation leads to dissociation of the axin/APC/GSK-3β complex. Beta-catenin phosphorylation is inhibited and it accumulates in the cytosol. The cytosolic beta-catenin translocates to the nucleus and binds to TCF/LEF factors. This results in the Wnt-responsive gene transcription. In Wnt “off state” (left part), Dsh dissociates from Fzd and Axin. APC and axin complex with GSK-3β. Beta-catenin is phosphorylated, dissociates from GSK-3β, migrates to the cytosol and is destroyed in the proteasome. Abbreviations: APC, adenomatous polyposis coli; Dsh, Disheveled; Fzd, Frizzled; GSK-3β, glycogen synthase kinase-3beta; LRP5/6, low density lipoprotein receptor-related protein 5/6; TCF/LEF, T-cell factor /lymphoid enhancer factor.

The Wnt/beta-catenin pathway (Figure 1) plays an important role in embryonic development and cell fate and its dysregulation is implicated in numerous pathological processes such as carcinogenesis (Moon et al., 2002, 2004; Nusse, 2005; Clevers, 2006). In the absence of Wnt activation (“off state”), cytosolic beta-catenin is phosphorylated by GSK-3beta. APC and Axin combine with GSK-3beta and beta-catenin in the destruction complex. This leads to destruction of the phosphorylated beta-catenin, which is degraded in the proteasome. In the presence of Wnt activation (“on state”), Wnt ligands bind both Fzd and LRP5/6 receptors. Dsh binds to axin, which prevents the GSK-3beta phosphorylation of beta-catenin. Then, beta-catenin accumulates in the cytosol, translocates to the nucleus and ultimately binds to LEF/TCF co-transcription factors. This leads to the transcription of Wnt-responsive genes including PPAR beta/delta, Axin-2, cyclin D1, CD44, c-myc, and Cox2 (He et al., 1998; Shtutman et al., 1999; Angers and Moon, 2009).

## Peroxisome proliferator-activated receptor (PPARs)

PPARs alpha, beta/delta, and gamma are ligand-activated transcriptional factor that belong to the nuclear hormone receptor superfamily. They heterodimerize with the retinoid X receptor and bind to peroxisome proliferator response elements which are specific regions on the DNA of target genes. PPAR alpha has been first discovered and characterized as a rodent hepatocarcinogen that causes proliferation of peroxisomes. PPAR alpha is a key regulator of lipid metabolism and is activated under conditions of energy deprivation. It is highly expressed in tissues that oxidize fatty acids at a rapid rate and activates numerous genes involved in cellular fatty acid uptake, mitochondrial fatty acid beta-oxidation and lipoprotein metabolism (Desvergne and Wahli, 1999; Watanabe et al., 2000). PPAR alpha is activated by endogenous ligands or by synthetic ligands such as fibrates which are used in the treatment of hyperlipidemia.

PPAR gamma is expressed in various cellular types, including adipose tissues, immune cells, and brain cells (astrocytes and microglia). It regulates glucose homeostasis, insulin sensitivity, lipid metabolism, cell fate, immune responses, inflammation, and cardiovascular function (Elbrecht et al., 1996; Fajas et al., 1997; Desvergne and Wahli, 1999). PPAR gamma is dysregulated in various diseases including obesity, type 2 diabetes, cancers and atherosclerosis. PPAR gamma agonists thiazolidinediones (TZDs) are insulin sensitizing molecules and some of them are used in the type 2 diabetes treatment (Picard and Auwerx, 2002; Rangwala and Lazar, 2004). TZDs act on the promoters of GLUT2 and beta- glucokinase in pancreatic beta-cells and liver. PPAR gamma controls circadian variations in blood pressure and heart rate through the clock gene *BMAL1* (Wang et al., 2008) and plays a role in the occurrence of instabilities in systems that thermodynamically behave far-from-equilibrium (Prigogine and Nicolis, 1971; Lecarpentier et al., 2010). PPAR gamma induces neuroprotective and anti-inflammatory effects (Kapadia et al., 2008; Gray et al., 2012; Katsouri et al., 2012). PPAR gamma ligands induce beneficial effects in many NDs such as amyotrophic lateral sclerosis, Parkinson's disease, Alzheimer's disease, Huntington's disease, multiple sclerosis and stroke.

## Crosstalk between canonical Wnt/beta-catenin signaling and PPAR gamma

### PPAR gamma agonists induce beta-catenin inhibition

PPAR gamma and the Wnt/beta-catenin pathway have been shown to behave in an opposite manner (Gerhold et al., 2002; Girnun et al., 2002a,b; Sharma et al., 2004; Takada et al., 2009; Lu and Carson, 2010). The functional interplay between PPAR gamma and Wnt/beta-catenin signaling implicates the TCF/LEF binding domain of beta-catenin and a catenin binding domain (CBD) within PPAR gamma. (Liu et al., 2006). Heterozygous loss of PPAR gamma increases the beta-catenin level in a genetic model of colon cancer. Thus, PPAR gamma can inhibit beta-catenin (Girnun et al., 2002b). Conversely, beta-catenin can directly interact with PPAR gamma and RXR alpha (Xiao et al., 2003; Jansson et al., 2005; Liu et al., 2006). TZDs PPAR gamma agonists repress beta-catenin-dependent transcription (Lu and Carson, 2010). Activation of PPAR gamma induces the proteasomal degradation of beta-catenin in cells that express an APC-containing destruction complex, although oncogenic beta-catenin inhibits the expression of PPAR gamma target genes (Liu et al., 2006). PPAR gamma inhibits osteoblastogenesis, promotes adipogenesis and suppresses the Wnt/beta-catenin pathway during adipogenesis (Moldes et al., 2003; Takada et al., 2009). TZDs induce a reduction in the levels of cytoplasmic beta-catenin in 3T3L1 adipocytes (Gerhold et al., 2002) and in hepatocytes (Sharma et al., 2004). Conversely Wnt/beta-catenin signaling activation inhibits PPAR gamma and leads to osteogenesis (Takada et al., 2009).

### Inhibition of Wnt/beta-catenin pathway induces activation of PPAR gamma

Deactivation of the Wnt/beta catenin pathway and activation of PPAR gamma are observed in arrhythmogenic right ventricular cardiomyopathy (ARVC) (Garcia-Gras et al., 2006; Djouadi et al., 2009). Gamma-catenin (or plakoglobin), which presents structural similarities with beta-catenin (Moon et al., 2002), translocates to the nucleus, competes with beta-catenin and inhibits Wnt/beta-catenin signaling through TCF/LEF transcription factors (Zhurinsky et al., 2000). This enhances adipogenesis, thus summarizing the phenotype of the human ARVC (Garcia-Gras et al., 2006; Djouadi et al., 2009).

## Alzheimer's disease (AD) generalities

**Figure 2:**
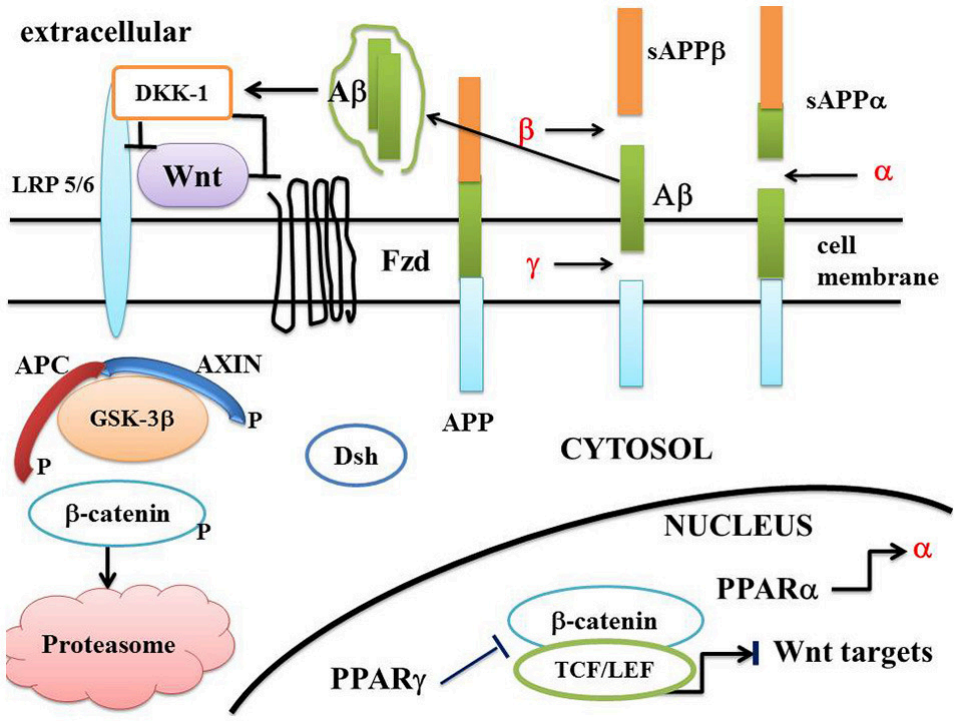
**A schematic model of the Wnt/beta-catenin pathway in Alzheimer disease**. In AD, the “on state” of canonical Wnt/beta-catenin pathway is interrupted at two potential levels. Firstly, extracellular Aβ activates DKK1 which inhibits the interaction between Wnt and LRP 5/6. Binding of Wnt to Fzd is suppressed and Dsh dissociates from Fzd/Axin and migrates to the cytosol. The recruitment of the destruction complex to the plasma membrane is suppressed. Inactivation of Dsh leads to stimulation of GSK-3β. Thus, APC and AXIN interact with GSK-3β and beta-catenin (Wnt “off state”). Beta-catenin is phosphorylated by GSK-3β. The destruction complex AXIN/APC/GSK-3β is activated and enhances the destruction process of beta-catenin in the proteasome. Secondly, PPAR gamma which is activated by agonists can inhibit the beta-catenin/TCF/LEF complex in the nucleus, thus inhibiting the transcription of Wnt target genes. Amyloid precursor protein (APP) can be cut into various fragments by α, β, and γ secretases. PPAR α activates the transcription of α secretase. Abbreviations APC, adenomatous polyposis coli; Dsh, Disheveled; Fzd, Frizzled; GSK-3β, glycogen synthase kinase-3beta; LRP5/6, low density lipoprotein receptor-related protein 5/6; TCF/LEF, T-cell factor /lymphoid enhancer factor; sAPPα, soluble APPα ectodomain; sAPPβ, soluble APPβ ectodomain; APP, Amyloid precursor protein; α, β, and γ, α, β, and γ secretases; Aβ, amyloid β peptide; DKK1, Dickkopf-related protein1.

The molecular mechanisms underlying the pathophysiology of AD are still not fully understood. AD is characterized by the deposition of extracellular Abeta plaques (Abeta) and the formation of NFTs in the CNS. NFTs contain the aggregated hyperphosphorylated microtubule-associated protein (MAP) tau. Abeta plaques induce neural dysfunction and cognitive impairment (Mattson, 2004; Buée et al., 2010; Mayeux and Stern, 2012). There is an extracellular beta-amyloid deposition in specific regions of the brain which contains Abeta peptides. These protein fragments derive from proteolytic cleavage of the amyloid precursor protein (APP), a membrane protein (Hicks et al., 2013; Nalivaeva and Turner, 2013; Figure 2).

The hypothesis of AD amyloid cascade has been formulated by Hardy and Higgins (Hardy and Higgins, 1992). In AD there is an accumulation of extracellular amyloid aggregates of amyloid β peptide (Abeta) (or senile plaques) and intracellular neurofibrillary tangles of hyperphosphorylated tau protein. Abeta oligomers are toxic and accumulate particularly in the neocortex and hippocampus (Sisodia and Gallagher, 1998). APP is a type 1 integral membrane protein with three isoforms (Sandbrink et al., 1996). There are two proteolytic pathways for APP processes. The first one pathway, the amyloidogenic, involves the sequential cleavage of APP by β- and γ-secretases, leading to the release of a soluble ectodomain sAPPβ and Aβ peptide (Zhang et al., 2012). The second pathway, the non-amyloidogenic pathway, involves the α-secretase cleavage of APP (Allinson et al., 2003) and precludes Abeta formation. Abeta forms dimers and higher level oligomers which cause neuronal death. Abeta aggregates with other proteins to form amyloid plaques. Solube sAPPα ectodomain has neuroprotective properties (Figure 2).

Under physiological conditions, Abeta is released into the interstitial fluid of the brain. This improves the synapse function and neuronal activity (Palop and Mucke, 2010). In AD, Abeta contributes to AD development (Mattson, 2004; Wan et al., 2014a). Abeta is associated with progression of the disease. Its accumulation is thought to be responsible for neuroinflammation and to contribute to the AD pathophysiology. This results in CNS damages associated with impairment of hippocampal synaptic plasticity, progressive loss of the cognitive function and long-term depression (Mattson, 2004; Shankar et al., 2008; Cerpa et al., 2010; Palop and Mucke, 2010).

### The canonical Wnt/beta-catenin pathway in AD

Wnt pathways have been shown to be involved in several neuronal processes during embryonic development: synaptic differentiation, the function of neuronal circuits (by controlling neuronal differentiation), dendrite development, synaptic function, and neuronal plasticity (Rosso and Inestrosa, 2013). Wnt proteins participate in the remodeling of pre- and post-synaptic regions. Wnt proteins are constantly released in the brain to maintain the basal neural activity (Oliva et al., 2013b). Wnt is involved in adult neurogenesis and protects excitatory synaptic terminals from Abeta toxicity. There is a relationship between the loss of Wnt signaling and the neurotoxicity of Abeta in AD (Inestrosa et al., 2012). An endogenous Wnt-3a ligand prevents the toxic effects induced by Abeta in rat hippocampal neurons and protects hippocampal neurons from apoptosis induced by Abeta. The Wnt pathway is able to overcome the increase in GSK-3beta and tau phosphorylation and the decrease in cytoplasmic beta-catenin (Alvarez et al., 2004). Overexpression of Fzd-1 increases the Wnt-3a-induced cell survival, while inhibition of Fzd-1 reverses the Wnt-3a-induced beneficial effects. In PC12 cells, Wnt-3a increases beta-catenin expression (Kawamoto et al., 2012). Numerous other studies have observed a downregulation of Wnt/beta-catenin signaling in the pathogenesis of AD. This is attested by either a decreased level of beta-catenin or increased activity of both GSK-3beta and Dickkopf-1 (DKK1), two inhibitors of Wnt signaling (Rosi et al., 2010; Clevers and Nusse, 2012; Inestrosa et al., 2012).

### Abeta represses the canonical Wnt pathway, while activation of Wnt signaling attenuates abeta neurotoxicity

Dysfunction of the Wnt pathway induced by Abeta has been reported in AD (Thies, 2011; Silva-Alvarez et al., 2013; Wan et al., 2014b). Hyperphosphorylated tau and Abeta induce neuronal death and are responsible for a decrease in the cognitive function and progressive loss of memory (Price et al., 1998). Active Abeta plaques are found in abundance in microglia and astrocytes (Akiyama et al., 2000). In AD, Abeta downregulates the Wnt pathway. This is accompanied by synapse alteration and neuron degeneration (Silva-Alvarez et al., 2013). Cellular damages induced by Abeta are reversed by inhibition of GSK-3beta (Li et al., 2013). In AD, Wnt signaling activation induces neuroprotective effects via Wnt-3a (Alvarez et al., 2004), Wnt-5a, and Wnt-7a (Quintanilla et al., 2005), attenuates deleterious effects induced by Abeta, and facilitates the behavior of hippocampal neurons (Alvarez et al., 2004; Cerpa et al., 2010; Silva-Alvarez et al., 2013). In rat hyppocampal neurons, activation of the Wnt pathway by Wnt-3a prevents the Abeta-induced toxic effects, induces a decrease in GSK-3beta and tau phosphorylation and increases the level of cytoplasmic beta-catenin (Alvarez et al., 2004).

### GSK-3beta, a negative regulator of the canonical Wnt pathway

GSK-3beta inhibition induces neuroprotection in a transgenic AD model and in hippocampal cultured neurons. In the hippocampus of AD patients, increased GSK-3beta helps decrease the beta-catenin level and increase tau phosphorylation and NFTs formation (Oliva et al., 2013a). GSK-3beta phosphorylates MAP tau leading to NFTs (Buée et al., 2010; Mendoza et al., 2013; Rosso and Inestrosa, 2013). In the hippocampus of AD patients, both the expression and activity of GSK-3beta are augmented (Hooper et al., 2008; Oliva et al., 2013b). In AD, increased GSK-3beta expression is linked to memory abnormalities (Hooper et al., 2007; Killick et al., 2014). Moreover, activation of GSK-3beta stimulates the APP cleavage (Phiel et al., 2003; Inestrosa and Varela-Nallar, 2014). Activation of Wnt signaling via GSK-3beta inhibition favors neuroprotection (Vargas et al., 2014).

### DKK-1, a negative regulator of the canonical Wnt pathway

DKK-1, a secreted glycoprotein, is favored by Abeta. Its expression is increased in AD. DKK-1 binds with LRP 5/6, blocks Wnt /Frd interaction and inhibits interaction with Wnt ligands (Mao et al., 2001; Figure 2). DKK-1 and Wnt ligands have two distinct sites on LRP 5/6. DKK-1 colocalizes with both hyperphosphorylated tau and GSK-3beta staining. Exposure of cultures to Abeta induces the expression of DKK1. DKK1 negatively modulates the Wnt pathway and activates the tau-phosphorylating GSK-3beta. An increase in DKK-1 is observed in the AD brain of humans and transgenic mice (Caricasole et al., 2004; Palop and Mucke, 2010; Rosi et al., 2010). DKK-1 silencing favors the phosphorylated form of GSK-3beta (Caricasole et al., 2004). DKK-1 is involved in synapse loss due to Abeta. DKK-1 antibodies suppress the synaptic loss due to Abeta in mouse brain (Purro et al., 2012). Inhibition of DKK-1 protects against Abeta-induced apoptosis. The secreted Wnt antagonist DKK-1 is required for amyloid beta-mediated synaptic loss (Caricasole et al., 2004; Rosi et al., 2010; Purro et al., 2012; Killick et al., 2014). The plasma cholesterol transport molecule, Apo-E4, induces inhibition of Wnt signaling in PC12 cells and indirectly increases DKK-1 expression, thereby enhancing Abeta toxicity (Caruso et al., 2006; De Ferrari et al., 2007; Donahue and Johanson, 2008).

Thus, activation of the Wnt pathway via Wnt ligands or inhibition of key negative regulators of the Wnt pathway, such as DKK-1 and GSK-3beta, are able to protect against Abeta neurotoxicity and to ameliorate cognitive performance in AD patients (Alvarez et al., 2004; Rosi et al., 2010; Shruster et al., 2011; Clevers and Nusse, 2012; Maguschak and Ressler, 2012a,b; Purro et al., 2012; Vargas et al., 2014). DKK1 contributes to the pathological cascade triggered by Abeta and is critically involved in tau phosphorylation.

### Lithium, an inhibitor of GSK-3beta

The GSK-3beta inhibitor, lithium chloride, regulates adult hippocampal progenitor development through Wnt pathway activation (Wexler et al., 2008). Lithium chloride enhances activation of Wnt signaling (Hedgepeth et al., 1997; Sinha et al., 2005; Galli et al., 2013). Rat neurons are protected from Abeta by lithium (Inestrosa et al., 2012). Lithium activates the Wnt signaling in cultured hippocampal neurons and induces a neuronal protection in AD transgenic mice. Abeta exacerbates the neuronal dysfunction caused by tau expression in a Drosophila model of AD and treatment of flies with lithium attenuates the effects of Abeta (Folwell et al., 2010). Lithium improves hippocampal cognitive functions and neurogenesis in APP mice (Fiorentini et al., 2010). Lithium or Wnt ligands in AD animal models or in primary hippocampal neurons attenuate Abeta toxicity by recovering beta-catenin levels (Fuentealba et al., 2004).

### Riluzole, an enhancer of canonical Wnt/beta-catenin signaling

Riluzole rescues glutamate alterations, cognitive deficits, and tau pathology associated with P301L tau expression (Hunsberger et al., 2015; Whitcomb and Molnar, 2015). Riluzole improves performance in the rTg (TauP301L) 4510 mouse model of AD. The TauP301L-mediated reduction in PSD-95 expression, a marker of excitatory synapses in the hippocampus, is rescued by riluzole. However, it has been shown that riluzole is an enhancer of Wnt/beta-catenin signaling in both HT22 neuronal cells and adult hippocampal progenitor cells (Biechele et al., 2010). This may partly explain the beneficial results induced by riluzole in AD mice. Conversely, riluzole has been approved for the treatment of amyotrophic sclerosis (ALS), a disease in which the Wnt/beta-catenin signaling is upregulated. It has been used in ALS due to its role in glutamate toxicity. Thus, riluzole induces various effects on the presynaptic inhibition of the glutamate release and the blockade of the voltage-gated sodium channel (Aggarwal and Cudkowicz, 2008). However, in ALS, riluzole presents a weak efficacy with prolongation of median survival by about 3 months (Bensimon et al., 1994; Lacomblez et al., 1996; Miller et al., 2003, 2012). This may partly explain the poor results obtained by using riluzole in ALS where the Wnt/beta catenin signaling is upregulated (Lecarpentier and Vallee, 2016). Wnt/beta-catenin signaling is downregulated in bipolar disorder and riluzole reduces symptoms in this disease (Gould and Manji, 2002; Pittenger et al., 2008; Valvezan and Klein, 2012). Similarly, by stimulating Wnt/beta-catenin signaling, riluzole could be an interesting target for AD treatment (Biechele et al., 2010).

### Other activators of the Wnt/beta-catenin pathway in AD: curcumin, huperzine A, M1 muscarinic receptors

Curcumin activates the Wnt/beta-catenin pathway through inhibition of GSK-3beta activity in APPswe transfected SY5Y cells and increases the translocation of beta-catenin to the nucleus (Zhang et al., 2011). In transfected cells treated with Curcumin, expression of GSK-3beta diminishes in a dose- and time-dependent manner. In AD brains, Huperzine A, an acetylcholine inhibitor, inhibits GSK-3beta, activates Wnt signaling and stabilizes the cytosolic beta-catenin level (Wang et al., 2011). M1 muscarinic receptor activation protects neurons from beta-amyloid toxicity (Farías et al., 2004; Caccamo et al., 2006).

Cannabidiol, a non-psychoactive marijuana component, has been shown to rescue PC12 cells from toxicity induced by Abeta peptide (Esposito et al., 2006). Cannabidiol inhibits hyperphosphorylation of tau protein in Abeta-stimulated PC12 neuronal cells and its effect is mediated through the Wnt/beta-catenin pathway. Taken together, all these data suggest a positive feedback loop between the canonical Wnt pathway and Abeta. Thus, activation of Wnt signaling could represent a promising target for the treatment of AD (Mao et al., 2001; Alvarez et al., 2004; Farías et al., 2004; Caccamo et al., 2006; Wexler et al., 2008; Boonen et al., 2009; Cerpa et al., 2010; Fiorentini et al., 2010; Shruster et al., 2011; Zhang et al., 2011; Inestrosa et al., 2012; Kawamoto et al., 2012; De Ferrari et al., 2014).

### Canonical Wnt/beta-catenin pathway and PPAR gamma behave in an opposite manner in AD

In the adult brain and under physiological conditions, the expression of PPAR gamma has been reported to be at a low level (Kummer and Heneka, 2008). Conversely in AD brains, both PPAR gamma protein levels (Kitamura et al., 1999) and PPAR gamma mRNA levels (de la Monte and Wands, 2006) have been shown to be elevated. Moreover, the Wnt pathway is downregulated in AD (Thies, 2011; Silva-Alvarez et al., 2013; Wan et al., 2014b). As indicated earlier, several studies have shown that PPAR gamma and the Wnt/beta-catenin pathway operate through an antagonistic manner (Gerhold et al., 2002; Moldes et al., 2003; Sharma et al., 2004; Garcia-Gras et al., 2006; Liu et al., 2006; Takada et al., 2009; Lu and Carson, 2010). Other diseases present a similar profile in terms of Wnt/beta-catenin /PPAR gamma signaling, i.e., ARVC, osteoporosis, cardiac hypertrophy, bipolar disorder, and schizophrenia (Lecarpentier et al., 2014). Thus, the high PPAR gamma levels observed in AD may contribute to inhibit the Wnt/beta-catenin pathway (Figure 2). Consequently, the rationale that leads to the use of PPAR gamma agonists to treat AD would seem to merit discussion.

### Inflammation in AD

PPAR gamma is able to induce anti-inflammatory effects and this leads to the hypothesis that PPAR gamma might be beneficial in CNS diseases presenting inflammatory processes, especially AD. The anti-inflammatory effects of PPAR gamma may be explained by the fact that PPAR gamma is able to inhibit several pathways by interacting directly on NFκB, AP-1, STAT1, and NFAT (Daynes and Jones, 2002; Pascual et al., 2005). Moreover, troglitazone can induce anti-inflammatory effects on neurons independently of its PPAR gamma activity (Nishijima et al., 2001). Pioglitazone has a low penetration through the blood brain barrier (Maeshiba et al., 1997), while rosiglitazone does not penetrate the blood brain barrier at all (Chalimoniuk et al., 2004; Risner et al., 2006). However, rosiglitazone improves cognition in AD patients (Risner et al., 2006). In AD, inflammatory processes in microglia and the presence of inflammatory molecules may generate neuronal loss, contributing to the progression of the disease (Tanzi and Bertram, 2005; Heneka and O'Banion, 2007). Interleukins, TNF, monocyte chemotactic protein-1 are present in microglia (Sly et al., 2001). INos is expressed in AD brain and contributes to inflammation (Vodovotz et al., 1996; Lee et al., 1999; Heneka et al., 2001b). Although PPAR gamma protein and mRNA levels are high, the anti-inflammatory effects of PPAR gamma agonists could nevertheless justify their use in AD.

### Dual effects of NSAIDs in AD

Non-steroidal anti-inflammatory drugs (NSAIDs) have been shown to delay AD onset and reduce the risk of AD development. *In vivo*, treatment of APP transgenic mice with NSAIDs (Ibuprofen: NSAID + choline esterase inhibitor) diminishes amyloid deposition (Lim et al., 2000, 2001). NSAIDs act directly on the generation of Abeta (Weggen et al., 2001). Ibuprofen inhibits GSK-3beta, stabilizes beta-catenin and reverses the decrease in Wnt signaling due to Abeta (Farías et al., 2005). NSAID inhibition of beta-catenin requires a high level of expression of PPAR gamma and its co-receptor RXR-alpha (Lu et al., 2005). Importantly, NSAIDs also activate PPAR gamma and inhibit inflammatory processes in the AD brain. This has been the basis for the use of PPAR gamma agonists in AD (Lehmann et al., 1997; Heneka et al., 2001a; Landreth and Heneka, 2001; Kielian and Drew, 2003; Yan et al., 2003; Landreth et al., 2008).

### PPAR gamma in AD

In AD trangenic mice, rosiglitazone attenuates learning and memory deficits and decreases Abeta-42 in their brain (Pedersen et al., 2006; Escribano et al., 2010). Memory and cognition are improved in AD patients treated by means of rosiglitazone. PPAR gamma agonists decrease the Abeta activation of microglia and prevent hippocampal and cortical neurons from death (Combs et al., 2000; Kim et al., 2002; Luna-Medina et al., 2005). PPAR gamma regulates inflammation of microglia due to beta-amyloid (Combs et al., 2000). PPAR gamma agonists prevent the beta-amyloid-stimulated expression of the cytokine genes interleukin-6 and TNF alpha. In the AD mouse (Tg2576) overexpressing APP, oral treatment for 4 months by pioglitazone decreases Abeta 40 (Yan et al., 2003). Rosiglitazone treatment of Tg2576 mice induces a reduction of Abeta 42 in the brain (Pedersen et al., 2006). In aged APP (V7171) transgenic mice, high doses of pioglitazone diminish microglial and astroglial activation and Abeta plaques (Heneka et al., 2005).

Other cellular mechanisms can explain the beneficial effects of PPAR gamma in AD. PPAR gamma modulates APP processing through a beta-secretase mechanism (Sastre et al., 2003, 2006). PPAR gamma increases clearance for Abeta (Camacho et al., 2004). In cultured cells, PPAR gamma overexpression diminishes Abeta production and increases APP ubiquitination (D'Abramo et al., 2005). PPAR gamma prevents iNos expression in the SNC (Heneka et al., 1999, 2000).

### Paradoxical interaction between Wnt/beta-catenin and PPAR gamma in AD

It has been shown that pioglitazone and rosiglitazone protect rat hippocampal neurons against Abeta-induced neurodegeneration. This results in inhibition of GSK-3beta, an increase of beta-catenin levels and translocation of cytoplasmic beta-catenin to the nucleus (Inestrosa et al., 2005). Moreover, PPAR gamma agonists have been shown to activate Wnt/beta-catenin signaling and induce neuroprotective effects on hippocampal neurons (Fuentealba et al., 2004). Furthermore, activation of the Wnt pathway through disheveled and axin induces the stabilization of the microtubule network, which results in an increase in neurite length and axonal caliber (Ciani et al., 2004). This outcome was similar to that observed in neurons treated by troglitazone and rosiglitazone (Inestrosa et al., 2005). It was concluded that PPAR gamma activation can prevent neuropathological effects induced by Abeta, and can induce a positive modulation of the Wnt pathway in neurons. Moreover, Wnt signaling might activate PPAR gamma through AMPK, Sirt1, and PGC1-alpha (Godoy et al., 2014). Taken together, these data suggest that activation of PPAR gamma leads to activation of the Wnt/beta-catenin pathway. This is largely inconsistent with numerous studies showing that these two systems work in an opposite manner (Gerhold et al., 2002; Girnun et al., 2002b; Moldes et al., 2003; Xiao et al., 2003; Sharma et al., 2004; Jansson et al., 2005; Garcia-Gras et al., 2006; Liu et al., 2006; Takada et al., 2009; Lu and Carson, 2010). Activation of the Wnt/beta-catenin pathway represses PPAR gamma and vice versa. Moreover, numerous studies have shown that Wnt activation improves the AD clinical status in humans and animal models. Beneficial effects observed in AD due to PPAR gamma agonists are due to their anti-inflammatory properties rather than through activation of the canonical Wnt signaling.

### PPAR alpha in AD

In AD, PPAR alpha and the biological effects of PPAR alpha agonists have been much less studied than PPAR gamma and PPAR gamma agonists. Importantly, expression of PPAR alpha has been shown to be reduced in AD, while that of PPAR gamma is high (de la Monte and Wands, 2006). PPAR alpha plays a pivotal role in lipid metabolism and anti-inflammatory processes. In many diseases, the expression of PPAR alpha generally varies inversely with that of PPAR gamma (Lecarpentier et al., 2014). Thus, in ARVD and cardiac hypertrophy, expression of PPAR gamma is high and expression of PPAR alpha is low, whereas the reverse has been observed in type 2 diabetes and hypertension (Finck et al., 2002; Feige et al., 2006; Djouadi et al., 2009).

Recently, it has been demonstrated that PPAR alpha is able to stimulate the degradation of APP via the ADAM-10 system (Corbett et al., 2015). Sequential amyloidogenic proteolysis of APP by beta-secretase and gamma-secretase generates pathogenic Abeta between residues 36 and 43. On the other hand, the non-amyloidogenic juxtamembrane APP cleavage by alpha secretase, i.e., a disintegrin and metalloproteinase 10 (Adam 10), precludes Abeta generation and results in APP clearance (Allinson et al., 2003). Mutations in the alpha secretase ADAM 10 are associated with increased Abeta generation and AD susceptibility (Suh et al., 2013). Activation of PPAR alpha upregulates transcription of the alpha-secretase gene. This shifts the APP processing toward the alpha-secretase pathway. In neurons, ADAM 10 overexpression diminishes Abeta in an AD mouse model (Postina et al., 2004). Impairment of the ADAM 10 synaptic process generates a sporadic AD model (Epis et al., 2010). In human AD, a decrease in the ADAM 10 function has been reported (Colciaghi et al., 2002, 2004; Marcello et al., 2013). Knockdown of PPAR alpha (but not PPAR gamma and beta/delta) decreases the expression of *Adam 10* and overexpression of PPAR alpha restores ADAM 10 expression in neurons of *Ppara* ^−/−^ mice (Corbett et al., 2015). Moreover, neurons null for PPAR alpha are deficient in ADAM 10. Lentiviral delivery of WT PPAR alpha to *Ppara* ^−/−^ neurons restores of expression of ADAM 10. The WY14643 PPAR alpha agonist increases ADAM 10 expression in hippocampal neurons. Activation of PPAR alpha induces the expression of Adam 10 and subsequent alpha-secretase proteolysis of APP. Upregulation of Adam 10 signaling represents an interesting therapeutic strategy against overproduction of Abeta. These findings suggest PPAR alpha could be a therapeutic target for reducing Abeta burden in AD (Corbett et al., 2015), especially as PPAR alpha exerts powerful anti-inflammatory effects and Wnt/beta-catenin activation upregulates PPAR alpha expression (Kozinski and Dobrzyn, 2013).

## Conclusion

Alzheimer disease represents the most common form of senile dementia and is highly neurodegenerative. This review emphasizes the contrasting behavior of Wnt/beta-catenin signaling and PPAR gamma in Alzheimer's disease. It has been observed that these two systems work in an opposite manner in numerous pathological situations. In AD, the Wnt/beta-catenin pathway is downregulated while PPAR gamma is upregulated. This is also the case in bipolar disease. The opposite situation has been observed, however, in several neurodegenerative diseases such as amyotrophic lateral sclerosis, Parkinson's disease, Huntington's disease, multiple sclerosis and Friedreich's ataxia. Logically, AD therapy should be based on the activation of the Wnt/beta-catenin pathway and the inactivation of PPAR gamma. However, numerous authors have reported that PPAR gamma agonists produce favorable effects in Alzheimer's disease, both in man and transgenic animal models, but this may well be due to their anti-inflammatory properties. From a pathophysiological point of view, activation of the Wnt system/beta-catenin pathway by lithium or riluzole (both enhancers of the Wnt/beta-catenin signaling) would seem a logical treatment. However, at the present time Wnt enhancers have only been used in certain AD animal models. As for PPAR alpha, interest in its pathophysiological role has recently been highlighted by enhancing the degradation of the APP through its action on the Adam 10 pathway. This type of therapy opens up new possibilities for the treatment of Alzheimer's disease.

## Author contributions

All authors listed, have made substantial, direct and intellectual contribution to the work, and approved it for publication.

### Conflict of interest statement

The authors declare that the research was conducted in the absence of any commercial or financial relationships that could be construed as a potential conflict of interest.

## Abbreviations

APC: adenomatous polyposis coli
AD: Alzheimer's disease
APP: amyloid precursor proteins
ARVC: arrhythmogenic right ventricular dysplasia/cardiomyopathy
CNS: central nervous system
DKK1: Dickkopf 1
Dsh: Disheveled
Fzd: Frizzled
GSK-3beta: glycogen synthase kinase-3beta
LRP5/6low: density lipoprotein receptor-related protein 5/6
MAP: microtubule-associated protein
NDs: neurodegenerative diseases
NFTs: neurofibrillary tangle
NSAIDs: non-steroidal anti-inflammatory drugs
PPAR: peroxisome proliferator-activated receptor
TZDs: thiazolidinediones.
